# Snaptron: querying splicing patterns across tens of thousands of RNA-seq samples

**DOI:** 10.1093/bioinformatics/btx547

**Published:** 2017-09-01

**Authors:** Christopher Wilks, Phani Gaddipati, Abhinav Nellore, Ben Langmead

**Affiliations:** 1 Department of Computer Science, Johns Hopkins University, Baltimore, MD, USA; 2 Center for Computational Biology, Johns Hopkins University, Baltimore, MD, USA; 3 Department of Biomedical Engineering, Johns Hopkins University, Baltimore, MD, USA; 4 Department of Biomedical Engineering, Oregon Health & Science University, Portland, OR, USA; 5 Department of Surgery, Oregon Health & Science University, Portland, OR, USA; 6 Computational Biology Program, Oregon Health & Science University, Portland, OR, USA

## Abstract

**Motivation:**

As more and larger genomics studies appear, there is a growing need for comprehensive and queryable cross-study summaries. These enable researchers to leverage vast datasets that would otherwise be difficult to obtain.

**Results:**

Snaptron is a search engine for summarized RNA sequencing data with a query planner that leverages R-tree, B-tree and inverted indexing strategies to rapidly execute queries over 146 million exon-exon splice junctions from over 70 000 human RNA-seq samples. Queries can be tailored by constraining which junctions and samples to consider. Snaptron can score junctions according to tissue specificity or other criteria, and can score samples according to the relative frequency of different splicing patterns. We describe the software and outline biological questions that can be explored with Snaptron queries.

**Availability and implementation:**

Documentation is at http://snaptron.cs.jhu.edu. Source code is at https://github.com/ChristopherWilks/snaptron and https://github.com/ChristopherWilks/snaptron-experiments with a CC BY-NC 4.0 license.

**Supplementary information:**

Supplementary data are available at *Bioinformatics* online.

## 1 Introduction

The Sequence Read Archive (SRA) is a repository of sequencing data containing over 12 petabases (Leinonen *et al.*, 2011). Archives like the SRA allow researchers to reproduce past studies, combine data in new ways, and leverage data that would otherwise be too expensive or difficult to generate. But there is no convenient way to pose scientific questions against the archives without first downloading and re-analyzing data.

Snaptron is a search engine for querying splicing patterns in large, pre-analyzed collections of human RNA sequencing (RNA-seq) samples. Snaptron lends valuable context and support to hypotheses related to splicing patterns in human. Snaptron’s query planner combines the strengths of different indexing strategies—R-trees, B-trees and term-document inverted indices—to rapidly answer queries (Supplementary Fig. S1). While past efforts have sought to enable querying of sequencing and expression data (Kolesnikov *et al.*, 2014; Petryszak *et al.*, 2016; Solomon and Kingsford, 2016), Snaptron is unique both in the breadth of splicing data it can query and in its ability to rapidly answer sophisticated questions.

## 2 Materials and methods

We first used Rail-RNA (Nellore *et al.*, 2015) to analyze archived human RNA-seq samples, as described previously (Collado-Torres *et al.*, 2017; Nellore *et al.*, 2016a,b). Rail-RNA outputs is a table summarizing evidence for exon-exon splice junctions across all samples. We also created tables detailing metadata for each sample. This is the source material for Snaptron as well as for the intropolis resource (Nellore *et al.*, 2016a). Snaptron also annotates each junction with: (i) gene annotation status (Supplementary Table S1), (ii) count of samples with one or more reads covering the junction and (iii) junction coverage statistics, such as sum and mean, summarized over all samples with evidence for the junction.

Snaptron user may query any of these four compilations of human RNA-seq samples: *SRAv1* contains 43M junctions called from 21 504 public samples from the SRA. *SRAv2* contains 81M junctions called from 44 427 public samples from the SRA. *GTEx* contains 29M junctions called from 9662 samples from the v6 GTEx data freeze. *TCGA* contains 37M junctions called from 11 284 samples from TCGA.

Users query splicing patterns of interest by specifying filters on genomic region (R), sample metadata (S), or other summaries calculated over the relevant samples (F). These can be combined, as denoted by abbreviations like R+F (filtered region query) or R+F+M (filtered region query with metadata constraint). Snaptron also distinguishes basic queries from high-level queries. High level queries combine many basic queries to answer more sophisticated questions. High level queries include *Junction Inclusion Ratio (JIR)*, used to rank samples according to the relative prevalence of different splicing patterns, *Percent Spliced In (PSI)*, a special case of JIR for alternatively spliced cassette exons, *Shared Sample Count (SSC)* for determining overall prevalence of a splicing pattern, and *Tissue Specificity (TS)*. Snaptron can handle groups of queries where the junctions returned are either the union or intersection of individual queries. Snaptron’s architecture and user interfaces are detailed in Supplementary Figure S2 and Supplementary Notes S1 and S2. Snaptron’s query performance is investigated in Supplementary Note S3 and Supplementary Figure S3.

## 3 Results

Supplementary Note 4 points to all software used for these results.

### 3.1 Assessing putative novel junctions

Snaptron’s junction calls were made without use of gene annotation, so it can assess prevalence of annotated or unannotated events without bias. We demonstrate this by partly recreating the Goldstein *et al.* (2016) study, which searched for unannotated cassette exons in Illumina RNA-seq data from 16 tissues. A cassette exon was called novel if neither extreme coincided with an annotated junction, but the exon was in an annotated gene. Goldstein *et al.* (2016) found 249 novel exons and validated 216 in a separate cohort.

To study these 249 exons using Snaptron, we posed shared-sample-count (SSC) queries that gathered evidence for the exons in the SRAv2 and GTEx compilations and scored exons according to the number of samples with evidence for the exon (details in Supplementary Note S5). Of the 249 putative exons, 236 (94.8%) occurred in both the SRAv2 and GTEx compilations. We found 204 of the 236 were validated by Goldstein *et al.* (2016), while the remaining 32 failed validation. The validated exons had significantly higher SSC than the others (Supplementary Fig. S4), indicating the SSC query is a rapid, in-silico method for measuring prevalence and reliability of a putative novel event.

Also, though the original study considered the 236 exons to be unannotaed, Snaptron results showed that 132 were annotated, most by the more inclusive SIBgenes (https://genome.ucsc.edu/cgi-bin/hgTrackUi? db=hg38&g=sibGene) and ACEview (Thierry-Mieg and Thierry-Mieg, 2006) tracks. Thus, Snaptron makes it easy to understand the annotation status of splicing events with respect to a wide range of annotations.

### 3.2 Assessing tissue specificity

In a repetitive element locus (REL) exonizaton event, part of the interspersed repeat is spliced into a surrounding gene as an exon. Darby *et al.* (2016) report numerous such events in human, including some specific to brain or blood. We used Snaptron to assess tissue specificity of five events where the spliced-in exon was not annotated. We used an SSC query to confirm the five events occur in both the SRAv2 and GTEx compilations (more than 39 samples in both cases). We then used a tissue specificity (TS) query to measure specificity of the five REL exons with respect to the more comprehensive GTEx compilation (details in Supplementary Note S6). Results showed all five exonization events were tissue-specific (Kruskal-Wallis *P*<1·10^−2^). In this way, Snaptron can measure a splicing pattern’s tissue specificity, a proxy for biological function.

### 3.3 Ranking samples according to splicing pattern

We performed an experiment modeled on Nellore *et al.* (2016a)’s analysis of the anaplastic lymphoma kinase (ALK) gene’s ALK^ATI^ variant isoform. ALK is mutated or aberrantly expressed in some cancers, notably in the form of the ALK^ATI^ variant, characterized by an alternative transcription initiation (ATI) site (Wiesner *et al.*, 2015). We used Snaptron to show the ALK^ATI^ variant and related EML4-ALK fusion can be found in non-cancer samples.

We used a junction inclusion ratio (JIR) query to rank samples according to how often the excised junctions (missing in ALK^ATI^) occurred relative to the junctions present in both spliceforms (details in Supplementary Note 7). The top 10 samples ranked by JIR match those reported by Nellore *et al.* (2016a), including unexpected melanocyte and macrophage samples. This shows how Snaptron can rank samples according to the relative prevalence of a splicing pattern, such as a splicing signature for a disease phenotype. Snaptron also supports a percent spliced in (PSI) query that adapts JIR to the common case of an alternatively spliced cassette exon.

### 3.4 Graphical user interface

**Fig. 1:**
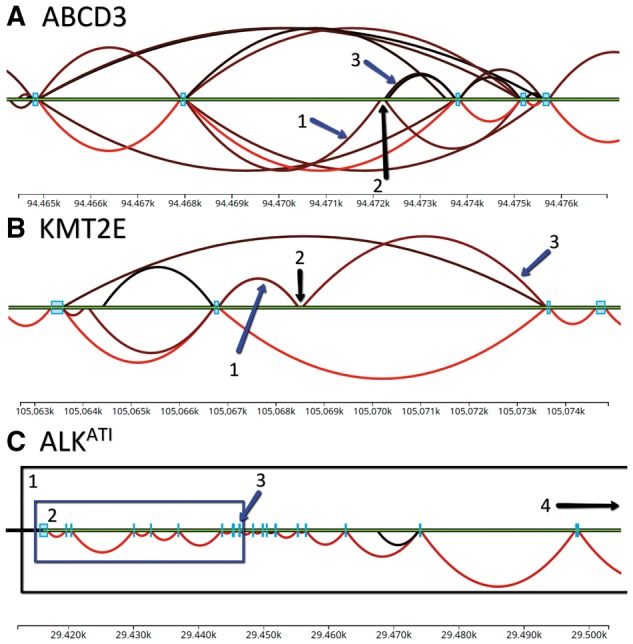
GUI screen captures related to experiments described in Results. Green horizontal lines indicate the genome. Arcs indicate exon–exon splice junctions. Arc colors indicate the number of samples having evidence for the junction, ranging from black (least support) to red (most). Annotated junctions are above the green line and unannotated junctions below. Blue rectangles are annotated exons. (**A**) Junctions matching Goldstein *et al.* (2016)’s prediction of a novel exon in the ABCD3 gene. A1 is the 5′ junction, A2 the novel exon, and A3 the 3′ junction; (**B**) KMT2E gene and unannotated junctions supporting a REL exonization event from Darby *et al.* (2016). B1 is the 5′ junction, B2 the REL exon, and B3 the 3′ junction; (**C**) ALK spliceforms studied by Wiesner *et al.* (2015) and Nellore *et al.* (2016a). C1 encloses the full length ALK transcript, C2 the ALK^ATI^ transcript incorporating only the last 10 exons (ALK is on the reverse strand, and so is laid out right-to-left), C3 points to the alternative initiation exon, and C4 points toward the upstream initiation site

We built a graphical user interface (GUI) to demonstrate how Snaptron queries can enable exploration and visualization of splice junctions across tens of thousands of samples (Fig. 1). A GUI user can (i) select a gene or region of interest, (ii) filter and color-code junctions according to summaries like shared sample count or average coverage and (iii) distinguish annotated from unannotated junctions. Supplementary Note 8 provides GUI links.

## 4 Discussion

Snaptron combines multiple indexing and database systems in a way that allows rapid queries, which can constrain flexible combinations of both structured interval and numeric data, and less structured textual metadata. This enables convenient new ways to explore and visualize splicing patterns over tens of thousands of individuals, measure the prevalence and reliability of putative novel splicing events, measure tissue specificity of possibly functional splicing patterns, and find samples with characteristic splicing patterns.

## Supplementary Material

Supplementary Figures
