# Supplementary material for: Snaptron: querying splicing patterns across tens of thousands of RNA-seq samples
Source: Bioinformatics. 2017 Sep 1;34(1):114–6. doi: 10.1093/bioinformatics/btx547 (PMC5870547; doi:10.1093/bioinformatics/btx547)
Supplement: Supplementary Figures [file snaptron-supplement_btx547.pdf]

Supplementary Information for
Snaptron: querying and visualizing splicing across tens of thousands of RNA-seq samples

Christopher Wilks, Phani Gaddipati, Abhinav Nellore, Ben Langmead

August 3, 2017

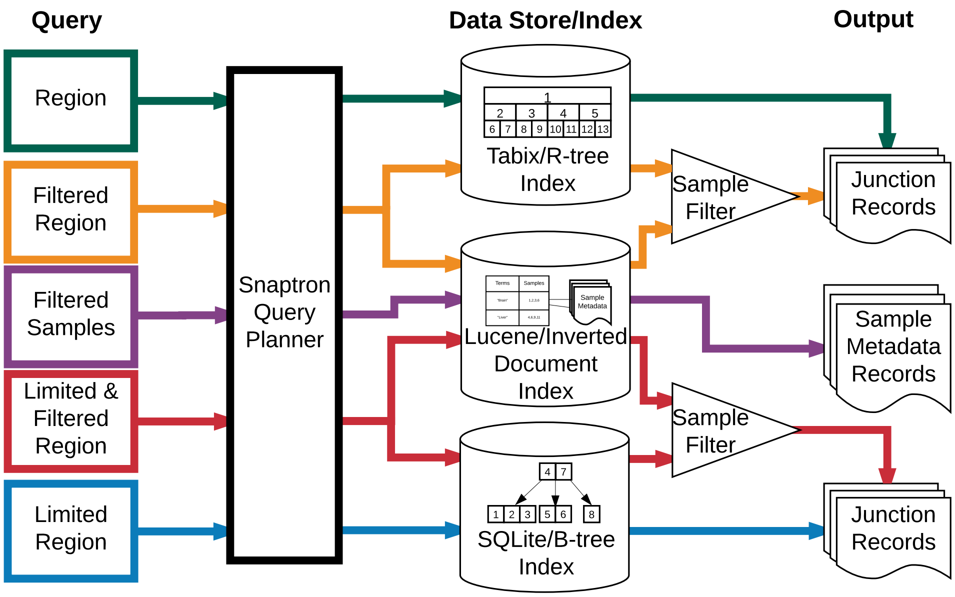

Supplementary Figure 1: The flow of each query through Snaptron and the type of output it produces. Colors correspond to those used for the queries in Supplementary Table 2.

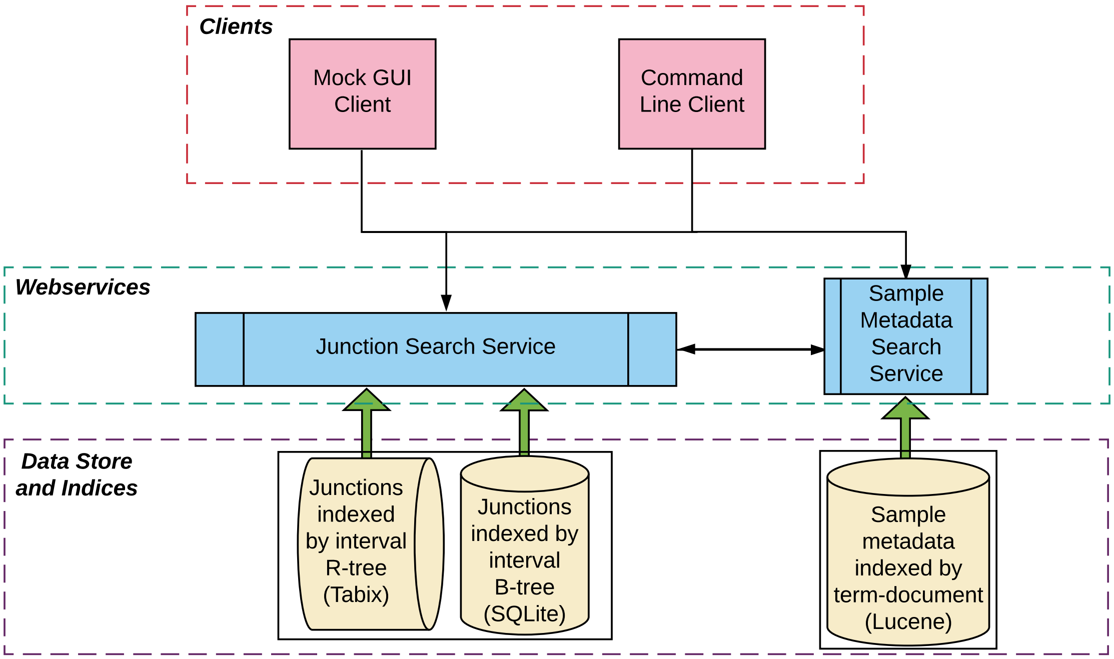

Supplementary Figure 2: The Snaptron architecture consists of three layers (from the bottom up) including data and associated indices (Tabix, SQLite, and Lucene), webservices and processing (Python), and finally the clients (NodeJS and Python). Queries issue from the clients and are processed by web services (black arrows) while responses flow back from the indices through the webservices to the clients (large, green arrows).

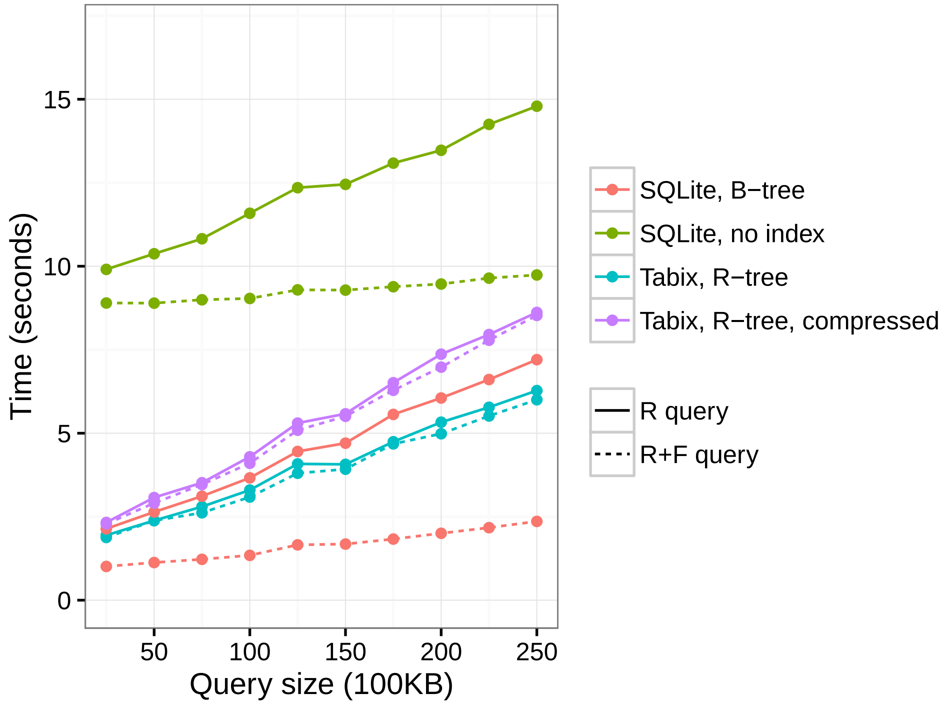

Supplementary Figure 3: Snaptron query wall-clock times for R and R+F queries of increasing size. The queries ask for all (for R) or some (for R+F) junctions overlapping an increasingly large prefix of chromosome 1. The region grows from a 2.5M-base prefix (leftmost) to 25M bases (rightmost) in 2.5M increments. The R+F constraint additionally requires all junctions returned to have `samples_count>=100`. The number of junctions returned by the R query range from 350K for the smallest (leftmost) to 1.5M for the largest (rightmost). The number of junctions returned by the R+F query range from 7.3K for the smallest to 28K for the largest. All data was uncompressed except where noted. Further experimental details are in Supplementary Note 3.

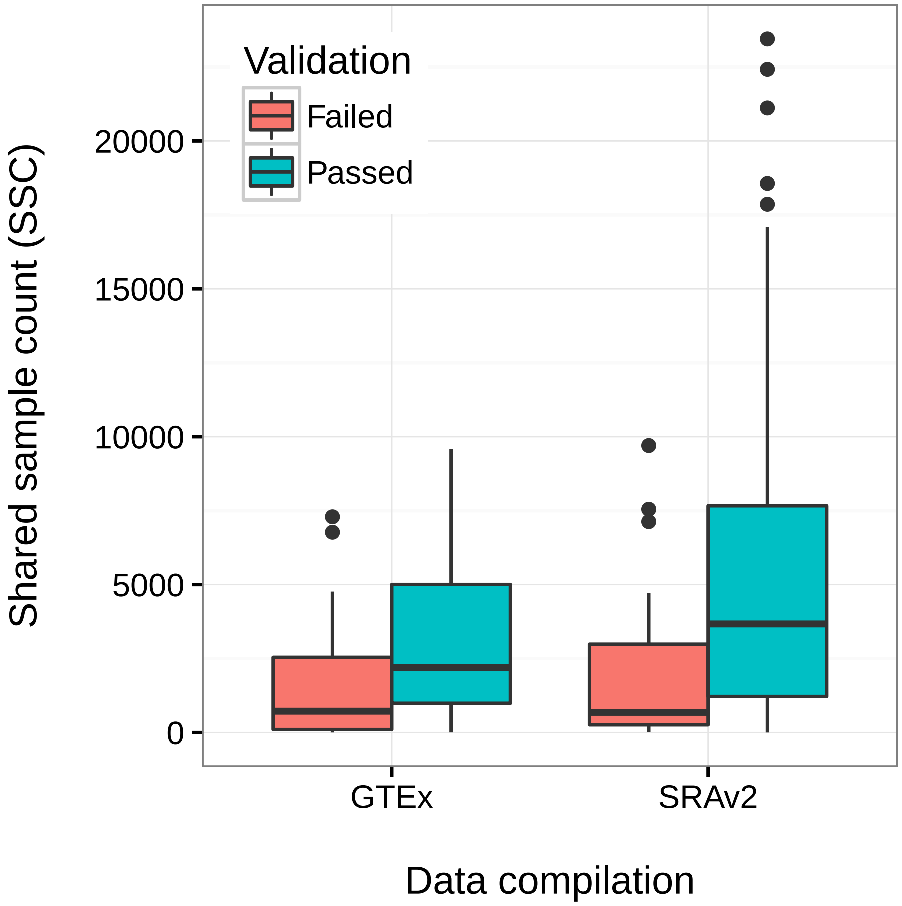

Supplementary Figure 4: Shared sample counts (SSCs) distinguishing novel cassette exons that Goldstein *et al.* (2016) validated in a separate cohort versus those that failed validation. Higher SSC correlates with greater probability of validating. For GTEx, Wilcoxon rank-sum p = 2e-04. For SRAv2, Wilcoxon rank-sum p = 1e-05.

Short Name	Description	Reference Build
Acembly	AceView gene models constructed from cDNA by Danielle and Jean Thierry-Mieg at NCBI, using their AceView program	HG19
ccdsGene	Human genome high-confidence gene annotations from the Consensus Coding Sequence (CCDS) project	HG19, HG38
Gencode	ENCODE-related annotation of Human genes from manual curation, computational processing, and experimental approaches.	HG19 (v19), HG38 (v24)
knownGene	A set of UCSC gene predictions based on data from RefSeq, GenBank, CCDS, Rfam, and the tRNA Genes track	HG19, HG38
lincRNAsTranscripts	Human Body Map lincRNAs (large intergenic non coding RNAs) and TUCPs (transcripts of uncertain coding potential)	HG19, HG38
mgcGenes	The Mammalian Gene Collection (MGC) of full-length open reading frames (ORFs) in the genome.	HG19, HG38
refGene	The NCBI RNA reference sequences collection (RefSeq)	HG19, HG38
sibGene	Swiss Institute of Bioinformatics cDNA/EST-based gene predictions	HG19, HG38
vegaGene	Annotated genes from the Vertebrate Genome Annotation (VEGA) database (Human chr14, 20, 22 only)	HG19

Supplementary Table 1: Gene annotation sources. Descriptions are from the UCSC Table Browser track detail page or the Gencode website

Query	Description	Examples in command-line syntax	Accessible from
**Basic Queries**			
**Region (R)**	Retrieve all junctions lying within a specified genomic interval. A gene name can be given in place of an interval, in which case the interval is taken to be the annotated extents of the named gene. For each returned junction, Snaptron reports a histogram of coverage levels for that junction across all samples with non-zero coverage.	chr1:1-100000<br>ALK	WSI, CCLI
**Region+Metadata (R+M)**	Like a Region query but with an additional metadata constraint that limits which samples are considered.	ALK&study_description:cancer	WSI, CCLI
**Region+Filter (R+F)**	Retrieve all junctions lying within a specified genomic interval but with an additional constraint that might eliminate junctions. The filter can constrain (a) the total, median or average coverage of the junction across samples where it occurs, (b) the number of samples where the junction has occurred, (c) whether or not the junction appears in the Snaptron annotation, (d) the junction's length, i.e. the number of bases spliced out, (e) the junction's strand.	ALK&sample_count>20<br>ALK&annotated=0<br>ALK&length>1000&length<2000	WSI, CCLI
**Region+Filter+Metadata (R+F+M)**	Combining elements of a Region+metadata query and a Region+filter query.	ALK&length>1000&<br>length <2000&<br>tissue:Brain	WSI, CCLI
**Metadata (M)**	Returns full sample metadata for each sample matching the metadata field query ranked by the Term Frequency - Inverse Document Frequency (TF-IDF) score	library_layout:paired; RIN>8	WSI, CCLI
**High Level Queries**			
Junction Inclusion Ratio Rank (JIR)	Given two basic junction queries defining two groups of junctions, this returns the list of sample records ranked according to the Junction Inclusion Ratio (JIR) calculated between the two groups. Each returned sample record includes the sample's full set of metadata, total count for the junctions in the first and second groups in that sample, and the JIR for that sample.	See Supplementary Note 4.	CCLI
Percent Spliced In (PSI)	Given a cassette exon, returns the list of sample records ranked according to the exon's Percent Spliced In (PSI). PSI is the fraction of the time the exon is spliced into the surrounding gene. The cassette exon is defined by specifying two "inclusion" junctions, used when the exon is spliced in, and one "exclusion junction," used when the exon is spliced out. As with JIR, each returned record includes the sample's full set of metadata along with the PSI.	See Supplementary Note 4.	CCLI
Shared Sample Count (SSC)	Given two groups of junctions, returns the number of samples that have non-zero coverage for at least one junction in both groups. Each group is defined by a basic junction query.	See Supplementary Note 4.	CCLI
Tissue Specificity (TS, GTEx only)	Given a group of junctions, returns a tissue specificity table for the set. The group is defined using a basic junction query. The table is N rows by 2 columns, where each row corresponds to one of the 9,662 samples in the GTEx v6 compilation. The first column contains a presence/absence indicator: 1 if every junction in the group is covered in that sample, 0 if not. The second column encodes which of the 32 tissue types the sample comes from.	See Supplementary Note 4.	CCLI

Supplementary Table 2: Snaptron basic and high-level queries. Basic queries are given a color corresponding to the colors in Supplementary Figure 1.

Supplementary Note 1: Snaptron architecture

Constraining metadata. Metadata constraints narrow Snaptron's focus to only those samples with metadata matching or containing key phrases. If we think of the junction evidence as forming a matrix with junctions as rows and samples as columns, metadata constraints narrow the query's focus to a subset of the columns. A metadata constraint can be used on its own in a Metadata (M) query, or combined with a Region (R) query or Region + Filter (R+F) query. We call the latter two combinations R+M and R+F+M queries (Supplementary Table 2).

Snaptron uses the Lucene (Bialecki *et al.*, 2012) inverted indexing system to handle metadata constraints. Snaptron includes Lucene indices for each data compilation: SRAv1, SRAv2, GTEx v6 and TCGA. An index associates over fifty metadata fields with each sample. The exact fields depend on the data source. Some fields contain unstructured ("free") text and describe, for example, how the sample was prepared and sequenced or what was being studied. Others are semi-structured, using text labels to describe categorical variables, such as whether the reads are paired-end or the sample's tissue type. For example, the GTEx compilation includes a controlled-vocabulary field describing the tissue of origin, but the SRA compilations do not; that information can often be gleaned from other free text-fields, though sometimes with difficulty (Bernstein *et al.*, 2016). The Lucene index allows searching for key phrases in a metadata field.

Query planning. Snaptron's query planner determines the combination of index probes needed to service a query. Region (R), Region + Filtered (R+F), and Metadata (M) queries are each answered from a different index; R queries use the Tabix R-tree, R+F queries the SQLite B-tree, and M queries the Lucene inverted index (Supplementary Figure 2).

The situation is more complex when a query combines region and metadata constraints, as in R+M and R+F+M queries. Again thinking of a junctions-by-samples evidence matrix, queries combining R and M constraints are concerned with a subset of columns (M constraint) and a subset of rows (R constraint). Such a query might ask for all junctions in the KCNIP4 gene that appear in at least 10 brain samples.

Handling this query decomposes into a few tasks. *Column projection* determines which samples

satisfy the metadata constraint. If C denotes the full set of columns (samples), let $C' \subset C$ be the subset satisfying the constraint, determined by querying the Lucene index. *Row projection* determines which junctions (rows) satisfy the region constraint. If R denotes the full set of rows (junctions), let $R' \subset R$ be the satisfying subset, determined by querying the Tabix index. Once C' and R' are known, *submatrix filtration* determines the subset of R' satisfying the "at least 10" constraint. Submatrix filtration is concerned only with the $R' \times C'$ submatrix. Consequently, summaries calculated over the full rows or columns of the original matrix cannot be used here; new summaries must be calculated with respect to the submatrix.

To perform submatrix filtration, sample IDs returned by the Lucene query are converted to an Aho-Corasick automaton. The automaton performs setwise pattern matching on Snaptron's internal string representation of the matrix rows. Specifically, Snaptron stores a row as a comma-delimited string, with each field containing the concatenation of the sample ID and the read coverage of the junction in that sample. To save space, samples with 0 coverage are not included as fields. The automaton analyzes a single row by consuming the row string's characters one-by-one and signaling when it has encountered one of the selected columns by entering a special "match" state. Each such match contributes a non-zero entry to the $R' \times C'$ submatrix. Once the submatrix is formed, Snaptron re-calculates row-wise summaries (e.g. sum, average, median). Finally, if an attribute filter (F) was specified, it is evaluated with respect to the recalculated summaries to further narrow the list of returned junctions, completing submatrix filtration.

Higher-level queries. Snaptron supports three queries that we term "higher-level" (Supplementary Table 2) because each involves junction sets defined using sub-queries. The *shared sample count (SSC)* query returns the number of distinct samples with evidence for the co-occurrence of two junctions. As we show in the main text of the paper, this is useful for studying prevalence of putative novel exons. The query output is easily loaded into an R or Python session and analyzed or visualized to better understand the prevalence of a splicing pattern.

The *tissue specificity (TS)* high-level query uses the GTEx v6 compilation. The user specifies one or more groups of junctions using one or more region sub-queries, one sub-query per group. The

TS query returns an $N \times 2$ table, where the N rows correspond to all 9.6K samples from the GTEx project and the two columns correspond to (a) whether a junction from every group occurred in that sample, and (b) which of the 32 GTEx v6 tissue types the sample was derived from. This list can then be loaded into Python or R to assess tissue specificity.

The junction-inclusion ratio (JIR) high-level query scores each sample according to a particular overrepresented splicing pattern relative to another. The user specifies two groups of junctions using two region sub-queries. Call these groups A and B. The query calculates the normalized difference between coverage counts of the two groups across all samples containing the junctions. This is the "junction inclusion ratio" (JIR) suggested by Nellore *et al.* (2016), but with one added to the denominator:

$$\frac{(B - A)}{A + B + 1}$$

A and B represent the total coverage for the two groups of junctions in the sample. JIR ranges from -1 to 1. Ranking samples according to JIR reveals the degree to which a splicing pattern is specific to a particular kind of sample.

Examples for both the additional PSI and "intersection" functions in the CCLI are under the PSI and Intersection sections at:

- `https://github.com/ChristopherWilks/snaptron-experiments/tree/AppNote`

Supplementary Note 2: Snaptron interfaces

Snaptron provides the following interfaces:

- RESTful web service interface (WSI): handles query requests made by a user or by other Snaptron interfaces via HTTP 1.1. Results come in the form of lists of junctions and associated junction data, or lists of samples and associated sample metadata. Queries usually return within seconds.

- Client command-line interface (CCLI): handles both basic queries and high-level queries. High-level queries are decomposed and handled via one or more WSI queries.

- Web-based graphical user interface (GUI): allows a user to visualize splicing patterns across many samples for a given window of the genome. Supports basic queries for populating the window. Queries are decomposed and ultimately handled by the WSI.

- Complete server installation (Local): users can download the underlying Snaptron data and software, build local indices and compilations, and run a local Snaptron service for handling WSI queries. This is for advanced users who require rapid processing of high query volumes.

Users experienced with command-line tools may prefer the direct WSI interface, which has minimal software requirements and responds within seconds in most cases. Users willing to install the lightweight Python CCLI can additionally pose high-level queries. The CCLI can be called from wrapper scripts to compose complex analyses, as shown in our example scripts.

The GUI is useful for users concerned with a particular gene or genomic region. The GUI will display junctions in that region, distinguishing clearly between annotated and unannotated junctions and using colors to differentiate junctions that occur more or less widely in the dataset. After exploring in the GUI, users can switch to the CCLI or WSI to answer specific questions.

The version of the Snaptron GUI presented here is available at:

- `https://github.com/ChristopherWilks/snaptronUI/tree/AppNote`

The exact version of Snaptron used for the analyses presented in the paper are available at:

- https://github.com/ChristopherWilks/snaptron/tree/AppNote

Supplementary Note 3: Performance benchmarking

All tests were run on warm caches. Tests were run three times and the results averaged. The test machine was a custom built Intel Core i7-4790K CPU @ 4.00GHz with 32 GiBs RAM and three 7200 RPM hard disks configured as separate Ext4 filesystems without RAID. This is also the machine hosting the Snaptron web services and GUI, at the time of this writing.

Experiments using the uncompressed (faster) version of the Tabix required modifying the source code of the bgzip program, which is another component of the HTSLib 1.2.1 library containing Tabix. The modification sets the `compress_level=Z_NO_COMPRESSION` flag for the file handle being written. This affects only the compression level, not the overall file format; the output of the modified Tabix can still be used with the unmodified Tabix (Li, 2011).

As stated in the main text we find that the SQLite B-tree index is somewhat faster than Tabix when the two are queried from within the Snaptron Python software and the Tabix junction data file is compressed. This is almost certainly due to overhead added by the Snaptron Python code. When we compare SQLite and Tabix outside of Snaptron, without Snaptron's overhead, they perform very similarly to each other (data not shown).

Supplementary Note 4: Reproducibility

Scripts and data needed to reproduce the analyses presented in Results section of the main text are available at: `https://github.com/ChristopherWilks/snaptron-experiments/tree/AppNote`. That page also includes links to query scripts that use the PSI high-level query type as well as the conjunction query type.

Supplementary Note 5: Assessing putative novel junctions

The query constrained the reported junction's strand to match the strand of the enclosing annotated gene. Further, the query included the "either" modifier to ensure one end of the queried junctions would exactly match the flanking coordinate on either the 5′ or 3′ end.

For this analysis the exon from the SRGAP2B gene was not able to be lifted over from GRCh37 to GRCh38 and was therefore not analyzed.

Supplementary Note 6: Assessing tissue specificity

We first obtained coordinates for 5 PCR-validated REL exonization events in three genes (KCNIP4, KMT2E and GLRB). We used the shared sample count (SSC) high-level query to measure the prevalence of the events in the SRAv2 and GTEx collections. We noted that the samples studied by Darby *et al.* (2016), derived from the Stanley brain collection, were not present in these compilations. We also found that none of the junctions flanking the events were fully annotated, in agreement with Darby *et al.* (2016).

We then performed a Kruskal-Wallis rank sum test on the TS query result, using the presence/absence results as the data and the tissue-annotation results as the group labels. All rank sum tests yielded $P < 1 \cdot 10^{-9}$, indicating strong tissue specificity. For example, the REL exon we refer to as `GLRB_1` is present in 33% of the 1,409 samples labeled "Brain" but only 3% of other samples. Similarly, the REL exon `KMT2E_1` is present in 56% of the 102 samples labeled "Bone Marrow" but only 12% of other samples.

Supplementary Note 7: Ranking samples according to splicing pattern

We used Snaptron's high-level JIR query to rank samples in order according to the difference between the total coverage of ALK junctions downstream of the ATI versus the junctions upstream. The sets of upstream and downstream junctions are defined using R+F queries. We constrained the strand to be the same as that of the ALK gene and required that junctions lie within ALK's annotated boundaries. Also following Nellore *et al.* (2016), we postprocessed the JIR results to exclude samples with fewer than 50 total reads covering the ALK junctions. Whereas Nellore *et al.* (2016) distinguish between the ALK^{ATI} variant and the EML4-ALK fusion by integrating other assays, we do not make the distinction here.

Supplementary Note 8: Graphical user interface

The Snaptron GUI was built using the MeteorJS framework (`https://www.meteor.com`).

Links to working instances of the GUI fronting the four compilations described in the paper are at the end of the Snaptron User's Guide:

- `http://snaptron.cs.jhu.edu/`

The version of the Snaptron GUI presented here is available at:

- `https://github.com/ChristopherWilks/snaptronUI/tree/AppNote`

References

Bernstein, M. N., Doan, A., and Dewey, C. N. (2016). Metasra: normalized sample-specific metadata for the sequence read archive. *bioRxiv*, page 090506.

Bialecki, A., Muri, R., and Ingersoll, G. (2012). Apache Lucene 4. *Proceedings of the SIGIR 2012 Workshop on Open Source Information Retrieval*, pages 17–24.

Darby, M. M., Leek, J. T., Langmead, B., Yolken, R. H., and Sabunciyan, S. (2016). Widespread splicing of repetitive element loci into coding regions of gene transcripts. *Human Molecular Genetics*, page ddw321.

Goldstein, L. D., Cao, Y., Pau, G., Lawrence, M., Wu, T. D., Seshagiri, S., and Gentleman, R. (2016). Prediction and quantification of splice events from rna-seq data. *PloS one*, **11**(5), e0156132.

Li, H. (2011). Tabix: fast retrieval of sequence features from generic tab-delimited files. *Bioinformatics*, **27**(5), 718–719.

Nellore, A., Jaffe, A. E., Fortin, J.-P., Alquicira-Hernández, J., Collado-Torres, L., Wang, S., Phillips III, R. A., Karbhari, N., Hansen, K. D., Langmead, B., and Leek, J. T. (2016). Human splicing diversity and the extent of unannotated splice junctions across human rna-seq samples on the sequence read archive. *Genome Biology*, **17**(1), 266.
